# A Rare Case of Cemento-Ossifying Fibroma: A Case Report

**DOI:** 10.7759/cureus.38685

**Published:** 2023-05-07

**Authors:** Vedha Aravindan, Santhosh P Kumar, Senthil Murugan P, Murugesan Krishnan, Alladi Sneha

**Affiliations:** 1 Oral and Maxillofacial Surgery, Saveetha Institute of Medical and Technical Sciences, Chennai, IND

**Keywords:** maxillofacial surgery, ossifying fibroma, curettage, surgical enucleation, anterior maxilla, calcifying epithelial odontogenic tumor, pagets disease, fibrous dysplasia, fibro-osseous lesions, cemento ossifying fibroma

## Abstract

Benign fibro-osseous lesions are a group of pathological conditions characterized by the replacement of normal bone with cellular fibrous connective tissue that undergoes mineralization. The most common types of benign fibro-osseous lesions include fibrous dysplasia, ossifying fibroma, and osseous dysplasia. However, diagnosing these lesions can be challenging due to their overlapping clinical, radiological, and histological features, which can cause a diagnostic dilemma for surgeons, radiologists, and pathologists. One rare type of benign fibro-osseous lesion is the cemento-ossifying fibroma (COF), which is a definitive form of a benign fibro-osseous tumor that affects the craniofacial region, particularly the jaws (70%). Here, we present a case of COF in a 61-year-old female patient in the maxillary anterior region. Due to a clear distinction between the lesion and healthy bone, the lesion was treated with conservative surgical excision followed by curettage and primary closure. However, differential diagnosis of COF can be highly challenging for clinicians due to its overlapping features with other fibro-osseous lesions like Paget’s disease and fibrous dysplasia. Ossifying fibroma and fibrous dysplasia often present a histopathological, clinical, and radiological overlap. The post-operative follow-up after eight months was unpredictable, with a radiological picture showing the increased thickness of the frontal bone, parietal bone, and maxilla with obliteration of marrow spaces, alteration of the trabecular pattern with a cotton wool/ground glass appearance, and reduced maxillary sinus space. Proper evaluation and diagnosis of fibro-osseous lesions are necessary before arriving at a final conclusion. Cemento-ossifying fibroma in the maxillofacial skeleton is uncommon, and after eight months, the recurrence rate is rare. This case highlights the importance of considering COF as a differential diagnosis for fibro-osseous lesions in the maxillofacial region and the necessity for proper evaluation and diagnosis to determine the appropriate treatment plan and prognosis. In summary, the diagnosis of benign fibro-osseous lesions can be challenging due to their overlapping features, but early diagnosis and proper evaluation are essential for successful treatment outcomes. COF is a rare type of benign fibro-osseous lesion where other fibro-osseous lesions in the maxillofacial region should be considered as a differential diagnosis, and the necessary steps should be taken to confirm the diagnosis before arriving at a final conclusion.

## Introduction

The fibro-osseous lesions of bone comprise a heterogeneous group of disorders that include developmental, neoplastic, or reactive (dysplastic) lesions characterized by the replacement of normal bone by fibrous tissue that acquires mineralization. They encompass two major lesions: fibrous dysplasia and ossifying fibroma. Cemento-ossifying fibroma (COF) is a definite form of benign fibro-osseous tumor affecting the craniofacial region (70%), principally in the jaws [1,2]. Most individuals are diagnosed in their second or third decades of life [3,4], and it is more common in women than in men in the ratio of 4:1 [5]. Radiologically, cemento-ossifying fibromas manifest in numerous patterns depending on the degree of mineralization of the lesion. We present a case of COF in a 61-year-old female patient in the anterior maxilla, which is a rare site for its occurrence. The excision and curettage of the lesion were carried out under general anesthesia. Recurrence of COF is occasional; the follow-up of this case after eight months shows differences in the radiographic pattern suggestive of other fibro-osseous lesions.

## Case presentation

**Figure 1:**
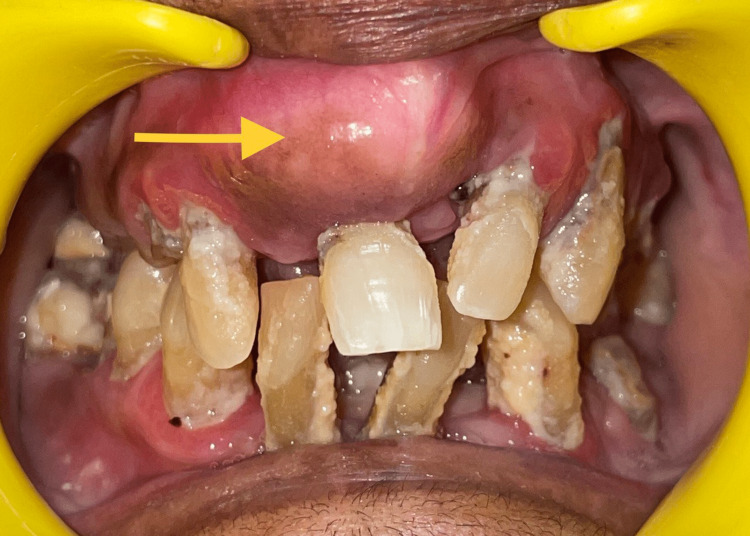
Pre-operative image The pre-operative image of bony mass in the anterior maxilla. The arrow mark depicts the bony enlargement in the anterior maxillary region.

A 61-year-old female patient reported to the Department of Oral and Maxillofacial Surgery at Saveetha Dental College, Chennai, Tamil Nadu. The patient's chief complaint was swelling over the upper front teeth region for the past five years. History revealed that the swelling was first noticed five years ago and had been causing mobility of the tooth for the past week (Figure 1). No history of pain was noticed, and there was no other swelling elsewhere in the body. The patient had a history of a thyroidectomy done two years ago; the patient was not on any medication, and there was no other medical history.

**Figure 2:**
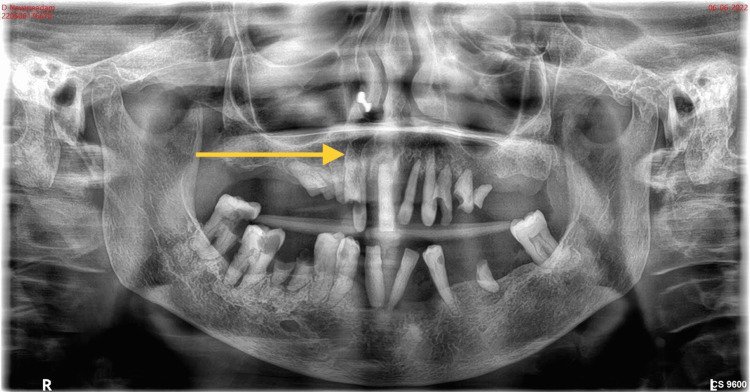
Pre-operative radiograph Represents the orthopantomogram showing the generalized horizontal bone loss in the upper arch and root stumps 13, 14, 15, 23, 24, 25, 36, and 46. Dental caries involving pulp in 47, 48, and 44 approximating pulp in 12 and 34. The arrow mark shows the generalized horizontal bone loss in the maxilla.

**Figure 3:**
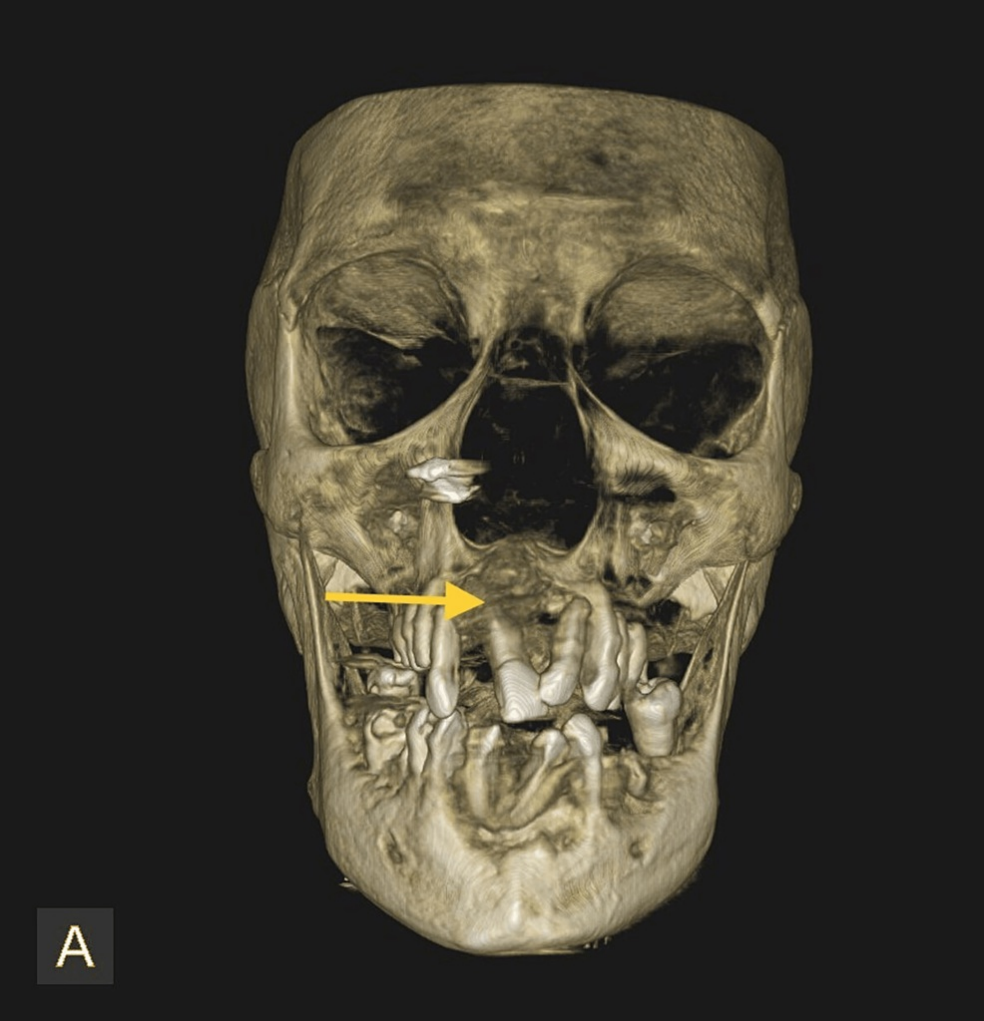
Pre-operative cone beam computed tomography (CBCT) Represents the 3D CBCT showing increased thickness of frontal bone, parietal bone, and maxilla. Obliteration of marrow spaces, alteration of trabecular pattern showing cotton wool/ground glass appearance. The arrow mark represents the displacement of teeth in the anterior maxilla.

Radiographic investigations were taken, and the orthopantomogram (Figure 2) showed a generalized horizontal bone loss in the upper and lower arch with root stumps 13, 14, 15, 23, 24, 25, 36, and 46; dental caries involving pulp in 47, 48, and 44; and dental caries approximating pulp in 12 and 34. Three-dimensional (3D) computed tomography (Figure 3) showed increased thickness of the frontal bone, parietal bone, and maxilla; obliteration of marrow spaces; and alteration of the trabecular pattern with a cotton wool/ground glass appearance. Displacement of teeth was seen in the anterior maxilla. Provisionally, the lesion was diagnosed as a fibro-osseous lesion or osteoma.

Surgical technique

**Figure 4:**
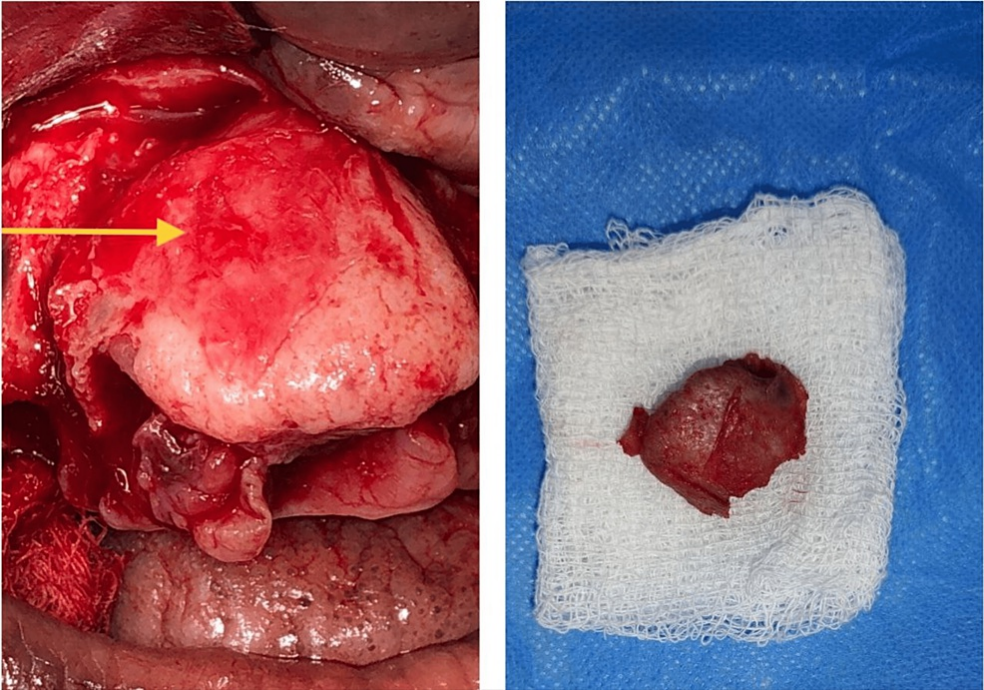
Intra-operative picture The arrow mark represents the intra-operative picture of exposure of cemento-ossifying fibroma (right) and excision of cemento-ossifying fibroma (left).

Under general anesthesia, all mobile teeth were extracted and a standard crevicular incision was made. The mucoperiosteal flap was raised, and there was a clear distinction between the lesion and healthy bone. The mass was excised (Figure 4) and sent for histopathological examination. The surgical site was curated, and primary closure was done with 3.0 polyglactin.

**Figure 5:**
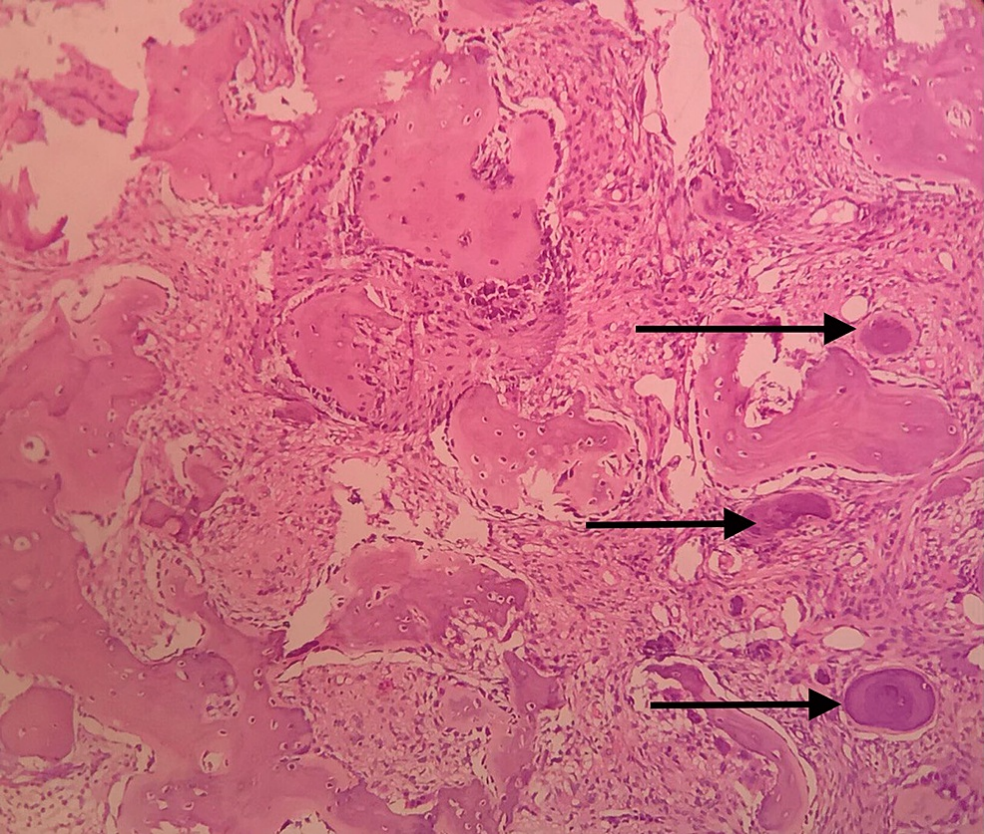
Histopathological section The section shows well-demarcated central fibro-cellular stroma with predominantly spindle and stellate-shaped cells with a basophilic hyperchromatic nucleus and minimal cytoplasm arranged in a random pattern. The arrow mark depicts basophilic calcification resembling cementum.

The histopathological picture (Figure 5) shows a well-demarcated central fibro-cellular stroma with predominantly spindle and stellate-shaped cells with basophilic hyperchromatic nuclei. There was evidence of numerous globular basophilic calcifications resembling cementum, suggesting cemento-ossifying fibromas.

**Figure 6:**
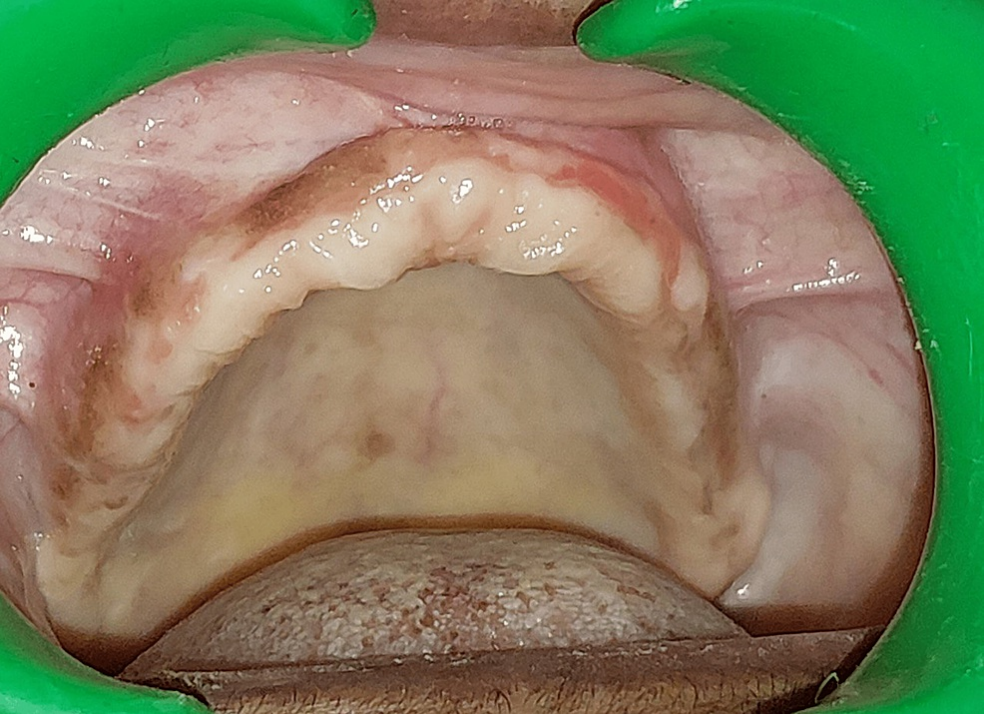
Post-operative intraoral image The intra-oral picture on a review after eight months shows satisfactory wound healing.

**Figure 7:**
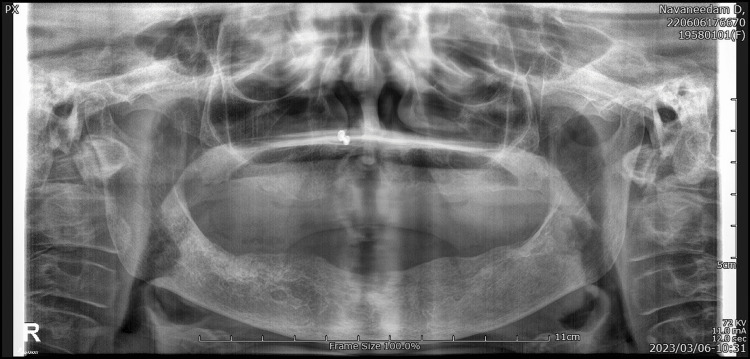
Post-operative radiograph Represents the post-operative orthopantomogram taken after eight months shows the completely edentulous upper and lower arch.

**Figure 8:**
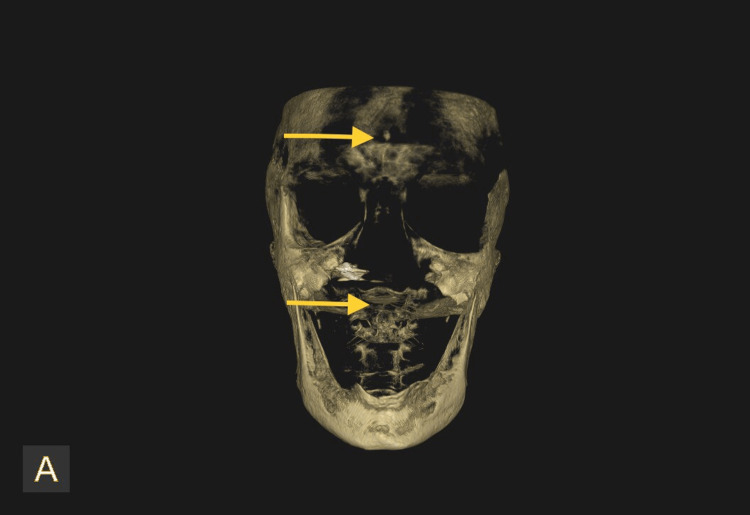
Three-dimensional view of CBCT after eight months Three-dimensional views of CBCT taken after eight months shows the increased thickness of frontal bone, parietal bone, and maxilla with obliteration of marrow spaces, alteration of trabecular pattern with cotton wool/ground glass appearance. Reduced maxillary sinus space is also evident.

Regular follow-up was done for eight months, and complete healing had taken place with no recurrence (Figure 6). The orthopantomogram is normal (Figure 7) with no evidence of calcification, but CBCT shows (Figure 8) increased thickness of the frontal bone, parietal bone, and maxilla with obliteration of marrow spaces and alteration of the trabecular pattern with a cotton wool/ground glass appearance.

## Discussion

Cemento-ossifying fibroma is a benign fibro-osseous tumor that is thought to have various controversies about its origin. It is believed to arise from the mesenchymal blast cells of the periodontal ligament and is composed of cementum, bone, and fibrous tissue [6,7]. Clinically, it appears as a slow-growing mass commonly located in the region of the mandibular premolars and molars, with a predominance in female patients and a peak incidence in the third and fourth decades of life [8]. The radiographic appearance of cemento-ossifying fibromas ranges from well-defined radiolucent to mixed radiolucent-radiopaque lesions, depending on the amount of bone and cementum-like material present. Histologically, COF is characterized by the presence of varying amounts of bone and cementum-like material, along with cellular fibrous connective tissue. The cementum-like material can appear in globular or laminated forms and may be calcified or non-calcified. A calcifying epithelial odontogenic tumor (CEOT) clinically resembles a cemento-ossifying fibroma but can be eliminated based on the age of occurrence of this tumor. A CEOT occurs between 8 and 92 years of age, with a mean age of 40 years, and its commonest site of occurrence is the molar area. It would be most commonly associated with an unerupted or impacted tooth. Radiographically, they appear similar initially, being radiolucent, but CEOT takes on a "honeycomb" or "driven snow" appearance as it advances [9]. The diagnosis of COF is quite challenging, as it requires appropriate clinical, radiological, and histopathological examination to rule out other fibro-osseous lesions and odontogenic tumors. The present case was reviewed after eight months and showed no fresh complaints or evidence of intraoral swelling (Figure 6). The orthopantomogram was normal (Figure 7) with no evidence of calcification, but cone beam computed tomography (CBCT) showed (Figure 8) increased thickness of the frontal bone, parietal bone, and maxilla with obliteration of marrow spaces and alteration of the trabecular pattern with a cotton wool/ground glass appearance [9].

The differential diagnosis of this could be highly challenging for the clinician due to the overlap between various fibro-osseous lesions, like Paget's disease, and fibrous dysplasia. Fibrous dysplasia is a benign fibro-osseous lesion that is also characterized by the replacement of normal bone by cellular fibrous connective tissue, leading to the formation of fibrous tissue and bone. Fibrous dysplasia and COF can have similar radiographic and histological features, making it difficult to differentiate between the two. However, fibrous dysplasia usually presents as a diffuse lesion involving the entire bone, whereas COF is usually a well-defined, localized lesion. On the radiological picture, fibrous dysplasia appears as a poorly differentiated lesion with a ground glass attenuation, contrary to cemento-ossifying fibroma. There have been reports in the literature of histological and radiological overlap between ossifying fibroma and fibrous dysplasia [10]. Steiner et al. suggested that ossifying fibroma is a subtype of fibrous dysplasia rather than a separate disease unit. In our case, the radiological and clinical pictures were inconclusive for surgeons, radiologists, and pathologists. Fibrous dysplasia also occurs in association with several endocrinopathies like hyperthyroidism, hyperparathyroidism, Cushing syndrome, acromegaly, and diabetes mellitus and is related to soft-tissue myxomas (Mazabraud syndrome). There are also syndromes associated with multifocal bone involvement, like polyostotic fibrous dysplasia and McCune-Albright syndrome. Polyostotic fibrous dysplasia is a rare genetic disorder that causes abnormal bone growth and bone lesions to develop in multiple bones, which cause deformities, fractures, and pain. McCune-Albright syndrome is a rare genetic disorder that causes a range of symptoms, including polyostotic fibrous dysplasia, café-au-lait spots on the skin, and endocrine abnormalities [10]. Paget's disease and cemento-ossifying fibroma are two distinct fibro-osseous lesions that can share overlapping clinical and radiological features, making diagnosis challenging. Paget's disease is a chronic disorder that results in the abnormal remodeling of bones and causes enlarged and weakened bones.

Radiographically, Paget's disease appears with a cotton-wool-like appearance and cortical thickening, while histologically, it displays disorganized and woven bone. Paget's disease may present with mixed radiolucent-radiopaque lesions; histological examination of the tissue can help differentiate between the two conditions. Paget's disease is typically a diffuse process that affects multiple bones, while cemento-ossifying fibroma is usually localized to a single bone [9,10]. Reduced maxillary sinus space is also evident in our case, which was unexpected. Another differential diagnosis for COF is ossifying fibroma. Ossifying fibroma is also a benign fibro-osseous lesion that can present with similar clinical, radiographic, and histological features as COF. However, ossifying fibroma is typically found in the mandible and has a higher incidence of recurrence than COF. Osseous dysplasia is another fibro-osseous lesion that can be difficult to differentiate from COF. Osseous dysplasia is a benign lesion that affects the jaws and is characterized by the replacement of normal bone by fibrous tissue and bone. Osseous dysplasia is usually asymptomatic and is more common in women than men [8,9]. However, osseous dysplasia typically presents as a diffuse lesion, whereas COF is usually a well-defined, localized lesion.

The treatment for COF could be complete excision and curettage. There are cases of cemento-ossifying fibromas that have been present for more than 30 years without any trouble or exacerbation [11]. In large lesions, the treatment involves ablative surgery with the additional challenge of replacing the excised tissue. Recurrence is uncommon and is a result of inadequate excision [6]. As there is a clear demarcation between the lesion and healthy bone, the treatment, in this case, is conservative surgical excision and curettage. The histopathological analysis of the specimen in the present case demarcated a central fibro-cellular stroma with predominantly spindle and stellate-shaped cells with basophilic hyperchromatic nuclei suggestive of a cemento-ossifying fibroma. But on follow-up after eight months, the radiological interpretation was unpredictable. Proper evaluation and diagnosis of the fibro-osseous lesion should be confirmed before concluding. Recent advances include the development of various bone grafting materials [12] and reconstruction with non-vascularized bone grafts [13]. But considering the age of the patient and the size of the defect, the site was closed primarily with absorbable suture material, which provides better wound healing [14]. The closure was done with 3.0 polyglactin, but there have been recent advancements in suturing techniques such as coating with silver nanoparticles [15], cyanoacrylate [16], and antibiotic-coated suture materials [17-19], which provide better wound healing.

## Conclusions

In conclusion, cemento-ossifying fibroma in the maxillofacial region, as presented in this report, is uncommon after eight months, and the differential diagnosis is quite challenging. The diagnosis of benign fibro-osseous lesions is highly challenging as they overlap in clinical, radiological, and histological presentations, thereby providing a diagnostic dilemma for surgeons, pathologists, and radiologists as the treatment plan and prognosis differ in each scenario. Clinical, radiological, and histopathological examinations should be carefully correlated before arriving at the final diagnosis, and adequate treatment protocols should be decided accordingly.
